# Near-Infrared Bioluminescence Imaging of Macrophage Sensors for Cancer Detection *In Vivo*

**DOI:** 10.3389/fbioe.2022.867164

**Published:** 2022-05-09

**Authors:** Giorgia Zambito, Gunja Mishra, Christopher Schliehe, Laura Mezzanotte

**Affiliations:** ^1^ Department of Radiology and Nuclear Medicine, Erasmus MC University Medical Center, Rotterdam, Netherlands; ^2^ Department of Molecular Genetics, Erasmus MC University Medical Center, Rotterdam, Netherlands; ^3^ Department of Immunology, Erasmus MC University Medical Center, Rotterdam, Netherlands,

**Keywords:** near-infrared bioluminescence, macrophage, cancer diagnostic, optical imaging, biosensor

## Abstract

Melanoma is an aggressive type of skin cancer with a poor prognosis after it gets metastasized. The early detection of malignant melanoma is critical for effective therapy. Because melanoma often resembles moles, routine skin check-up may help for timely identification of suspicious areas. Recently, it has been shown that the interplay of melanoma cells with the immune system can help develop efficient therapeutic strategies. Here, we leveraged engineered macrophages (BMC2) as cell-based sensors for metastatic melanoma. To perform dual-color bioluminescence imaging (BLI) *in vivo*, macrophages were engineered to express a green click beetle luciferase (CBG2) and a near-infrared fluorescent dye (DiR), and B16F10 melanoma cells were instead engineered to express a near-infrared click beetle luciferase (CBR2). Using real-time *in vivo* dual-color BLI and near-infrared fluorescence (FL) imaging, we could demonstrate that macrophages were able to sense and substantially accumulate in subcutaneous and metastatic melanoma tissues at 72 h after systemic injections. Together, we showed the potentiality to use optical imaging technologies to track circulating macrophages for the non-invasive detection of metastatic melanoma.

## Introduction

Detecting early-stage cancer is a promising avenue to enhance the effect of medical interventions and reduce cancer mortality (Etzion et al., 2003). Specifically, melanoma is an aggressive cutaneous type of cancer with an incidence that has been rapidly increasing in the past decade (Matthews et al., 2017). Nowadays, the identification of molecular markers together with histopathological assessment is more often used to guide prompt therapeutic decisions (Bianchini et al., 2007; Yang et al., 2020). These markers are melanoma mutations, gene polymorphisms, signaling receptors, and melanin pigment (Yang et al., 2020). In addition, melanoma is also one of the most immunologic malignancies associated with rapid infiltration of immune cells such as tumor-infiltrating T-lymphocytes and tumor-associated macrophages. The use of immunotherapies in the treatment of patients with metastatic melanoma has produced promising therapeutic advantages, increasing the overall survival of patients (Uhara 2019; Ralli et al., 2020). Thus, having early detection of metastatic tumor progression may significantly change the medical intervention in this type of tumor.

Here, we exploited monocytes as part of the innate immune system that are actively recruited into tumor tissues where they differentiate into tumor-associated macrophages (TAMs) and also accumulate in hypoxic areas (Burke et al., 2003; Yang et al., 2018). The accumulation of TAMs has also been demonstrated in primary lesions of melanoma and pulmonary metastases (Gajewski et al., 2013; Elpek et al., 2014; Pieniazek et al., 2018). The relative abundance of TAMs in melanoma ranges from 0 to 30%, and their density increases proportionally to tumor thickness (Hussein 2006). As a consequence of these natural properties, we decided to leverage light-emitting macrophages as a cellular sensor for the detection of subcutaneous and metastatic melanomas using a murine melanoma model as a proof-of-principle experiment. Engineered macrophages have previously been exploited as cell-based delivery platforms for breast cancer chemotherapeutics and also to shuttle oncolytic viruses specific to treat prostate cancer and related metastases (Muthana et al., 2013; Huang et al., 2021). Another seminal work proposed a cell-based “immunodiagnostic” system by using macrophages as pan-cancer cell sensors due to their ability to accumulate in breast tumors. In this study, macrophage sensors expressing a secreted form of Gluc (Gaussia luciferase) driven by an M2-like promoter were able to detect small tumors as small as 25–50 mm^3^ by real-time blood luciferase measurements (Aalipour et al., 2019).

However, the potential of macrophages as pan-cancer sensors has not been proved in other tumor models yet. Additionally, the description of macrophage dynamics and relative recruitment in the tumor site is still incomplete partly due to the scarcity of sensitive *in vivo* imaging detections (McCarthy et al., 2020; Su et al., 2020). In our previous work, we demonstrated that near-infrared dual-color bioluminescence imaging (BLI) is a sensitive method for the detection of two cell populations in deep tissues. However, the detection accuracy depends on the relative level of the expression of the two luciferases (Zambito et al., 2020; Zambito and Mezzanotte, 2021). Here, we exploited our previously developed method for the visualization of macrophage infiltration and for their accumulation in melanoma tissues *in vivo*. Therefore, we engineered BMC2 macrophages to express a near-infrared click beetle green luciferase mutant named CBG2 (*λ* = 680 nm) and B16F10 melanoma cells to express a near-infrared red click beetle luciferase mutant named CBR2 (*λ* = 740 nm) (Hall et al., 2018; Zambito et al., 2021). In addition to BL imaging, we also used a DiR (1,1′-dioctadecyl-3,3,3′,3′-tetramethylindotricarbocyanine iodide) near-infrared dye allowing non-invasive tracking of labeled macrophages after their systemic injection *in vivo* (Shan 2004; Eisenblatter et al., 2009; Liu and Wu 2016). Overall, this work provides a conceptual scenario for the use of engineered macrophages as a diagnostic sensor for cancer, and it provides proof-of-concept evidence for its successful application in clinically relevant murine melanoma models.

## Results

### Macrophages and Melanoma Cells Engineered to Express Luciferase Reporter Genes *In Vitro*

**FIGURE 1:**
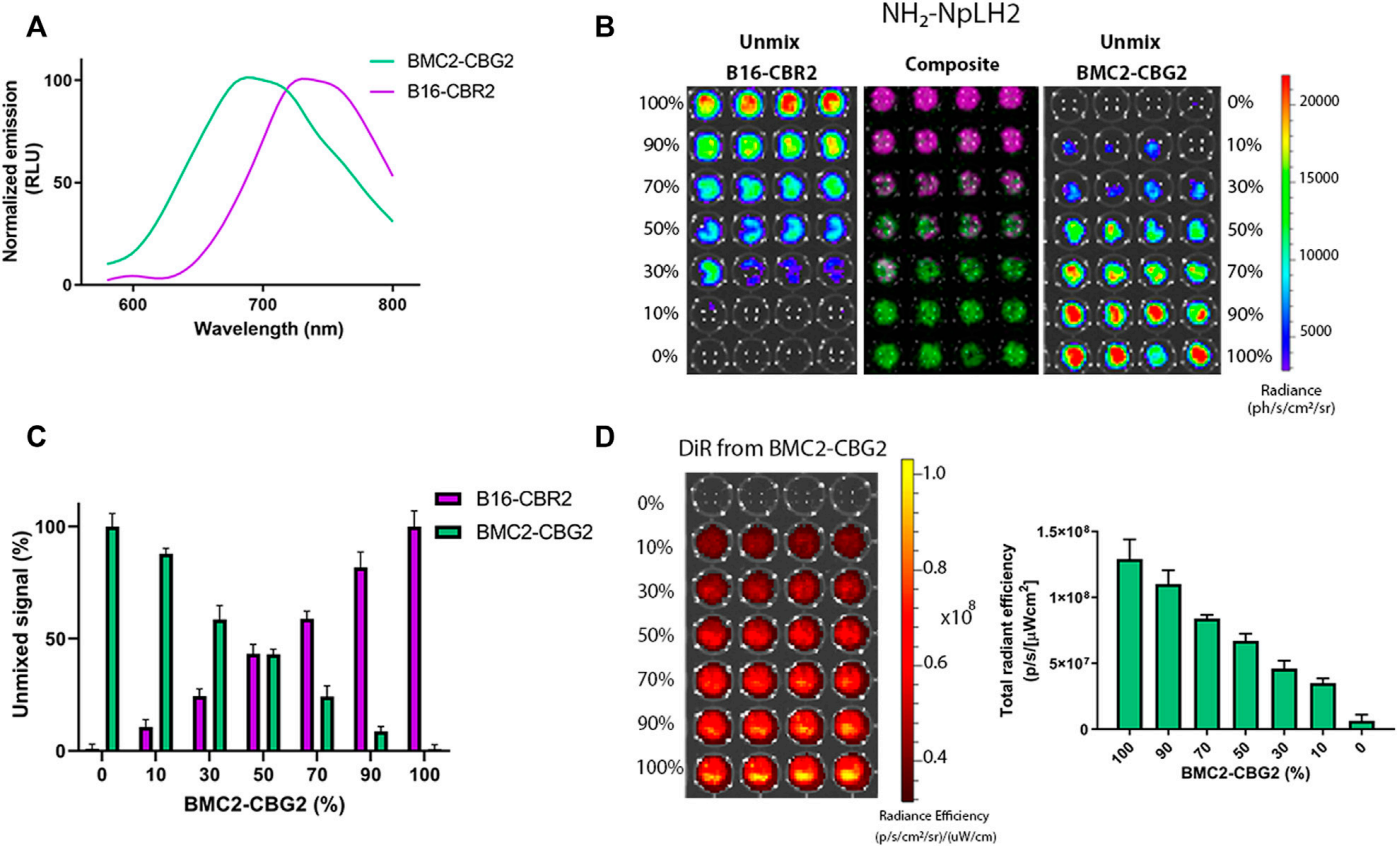
*In vitro* characterization and spectral unmixing of BMC2-CBG2 and B16-CBR2 luciferase-expressing cells. **(A)** Normalized bioluminescence emission spectra for BMC2-CBG2 and B16-CBR2 cells treated with NH_2_-NpLH2 luciferin as the luciferase substrate. Spectra were normalized to the peak emission for each click beetle mutants with the NH_2_-NpLH2 substrate. Spectra were acquired using an IVIS spectrum imager with the following settings: FOV C, f/stop = 1, medium binning, 30 s exposure time, and a range of bandpass filters ranging from 580 to 800 nm.**(B)** Spectral unmixing of B16-CBR2 and BMC2-CBG2 cells (*n* = 4 samples). Cells were mixed in various proportions ranging from 100 to 0% cell number of the total population. Quantification of the BL unmixed outputs for B16-CBR2 (magenta color) and BMC2-CBG2 (green color) registered after NH_2_-NpLH2 substrate administration. A black 96-well plate was spectrally imaged using a selected bandpass filter ranging from 580 to 800 nm by using an IVIS spectrum imager. **(C)** Quantification of the percentage of unmixed signals of B16-CBR2 and BMC2-CBG2 cells (*n* = 4 samples). Unmixed signals were normalized to 100% cell ratios with *p* < 0.0001 calculated by multiple comparisons and one-way ANOVA. Error bars represent ±SD. **(D)** Radiant efficiency was recorded for BMC2-CBG2 macrophages pre-labeled with the DiR near-infrared fluorescent dye. Cells were plated in a black 96-well plate in various proportions ranging from 0 to 100% of the total population. The bar graph shows the mean fluorescence intensity. Error bars represent ±SD (*n* = 4 samples). The plate was spectrally imaged using an IVIS imager system, and DiR filters were set at 710 nm for excitation and 760 nm for emission.

CBG2 and CBR2 luciferase mutants were engineered to have superior stability and red and near-infrared color-shift capability when combined with the NH_2_-NpLH2 luciferin substrate (Zambito et al., 2020). The macrophage cell line BMC2 was engineered to express the CBG2 luciferase mutant (BMC2-CBG2), and B16F10 melanoma cells were engineered to express CBR2 luciferase (B16-CBR2). To perform dual-color BLI and spectral unmixing *in vivo*, both BMC2-CBG2 and B16-CBR2 were seeded in a black 96-well plate, and living cells were spectrally imaged after the addition of NH_2_-NpLH2 luciferin on an IVIS spectrum imager. First, specific bioluminescent spectra of BMC2-CBG2 and B16-CBR2 cells were measured and saved in distinct spectral libraries. These libraries are useful to discriminate each luciferase contribution when BMC2-CBG2 and B16-CBR2 cells result colocalized in the same area of interest (Zambito and Mezzanotte 2021). As expected, the emission spectra registered a consistent red peak for CBG2 (*λ* = 680 nm) and a near-infrared peak for CBR2 (*λ* = 740 nm) when using NH_2_-NpLH2 luciferin (Figure 1A). Then, the spectral unmixing algorithm was applied for BMC2-CBG2 and pure B16-CBR2 cell mixture using selected pure libraries and setting bandpass filters ranging from 580 to 800 nm (Figure 1B). First, both cell types were cocultured at various ratios ranging from 100 to 0%, respectively, followed by spectral imaging in the presence of NH_2_-NpLH2 luciferin and subsequent unmixing (Figure 1B). As expected, the accurate spectral unmixing algorithm correctly classified the B16-CBR2-containing wells (unmix B16-CBR2) placed in the left panel of Figure 1B. With decreasing amounts of B16-CBR2 cells (magenta color in the composite figure), the CBR2-specific signal also gradually decreased toward the bottom of the plate. At the same time, BMC2-CBG2 cells (unmix BMC2-CBG2) were accurately classified in the right panel of Figure 1B. Here, the signal for BMC2-CBG2 cells (green color in the composite image) gradually decreased toward the upper end of the plate, which was in line with the increasing proportions of B16-CBR2 cells. The specific libraries were also used to quantify the photon flux of mixed BMC2-CBG2 and B16-CBR2 cell populations at various percentages between 100 and 0%. Quantification of the unmixed photon flux signals was normalized to 100% cell ratio and plotted in a bar graph revealing the linear correlation between the percentage of cells plated and the photons recorded (Figure 1C). To monitor higher accuracy BMC2-CBG2 cells *in vivo*, we labeled macrophages with a near-infrared fluorescent dye (XenoLight DiR, Perkin Elmer) to perform dual-optical imaging by bioluminescence (BL) and fluorescence (FL) imaging. To check the *in vitro* labeling efficiency, BMC2-CBG2 cells were prelabeled with DiR near-infrared dye and plated in various cell ratios ranging from 100 to 0% of the total volume in a black 96-well plate. Fluorescence imaging was performed by selecting DiR filters at 710 nm for excitation and 760 nm for emission. The radiant efficiency was calculated by subtracting the FL signal registered from unlabeled macrophages used as control. The fluorescence detected was found linearly proportional to the number of plated cells (Figure 1D). Additionally, to confirm that BMC2-CBG2 macrophages were successfully labeled with the DiR fluorescence dye, images of macrophages were taken at 24, 48, and 72 h after DiR treatment (Supplementary Figure S3A). Together, the *in vitro* results collected from BL and FL outputs validated that BMC2-CBG2 and B16-CBR2 cells could be further used for *in vivo* dual-color bioluminescence and fluorescence imaging.

### DiR-Labeled BMC2 Macrophages Detected in Subcutaneous Melanoma Tumors

**FIGURE 2:**
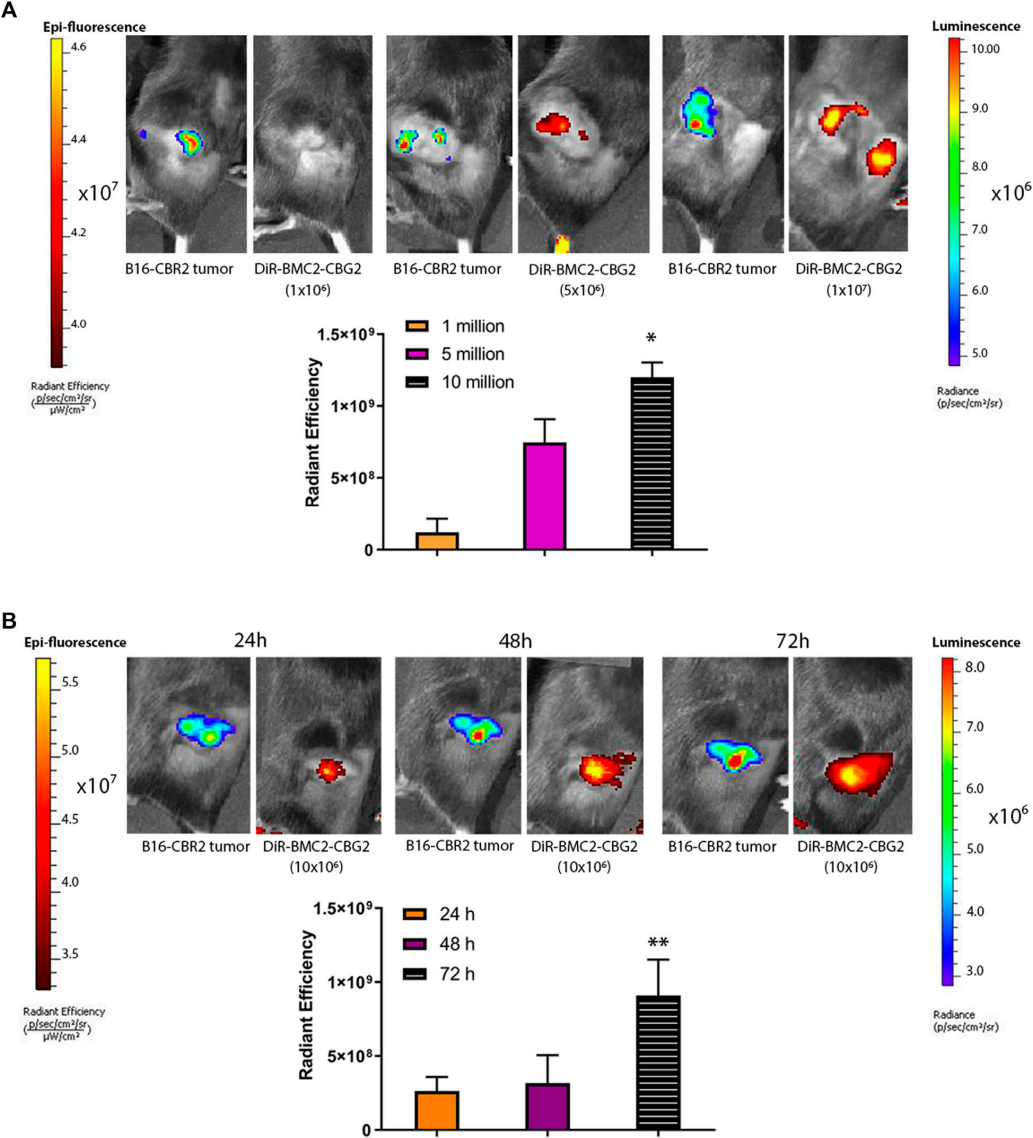
BMC2-CBG2 macrophages for subcutaneous melanoma detection. **(A)** Mice were transplanted with a subcutaneous B16F10 melanoma tumor model engineered to express near-infrared CBR2 click beetle luciferase (B16-CBR2). Bioluminescence photon fluxes represent the light emission for B16-CBR2 melanoma before BMC2-CBG2 administration. BL images were acquired 15 min after NH_2_-NpLH2 substrate injection. Before systemic injection, BMC2-CBG2 macrophages were pre-labeled with the DiR near-infrared dye (excitation 710 nm and emission 760 nm) at the amount of 1, 5, and 10 million per mouse (*n* = 3 mice). Respective radiant efficiencies were quantified and plotted. The bar graph shows the mean fluorescence intensity, and significance was registered for mean values of the 1-million group and mean values of the 10-million group. (*) *p* values (*p* < 0.05) were calculated by one-way ANOVA followed by Tukey’s test for multiple comparisons. Error bars represent ±SD. **(B)** Fluorescence values of BMC2-CBG2 macrophages pre-labeled with the DiR near-infrared dye (filters selected for excitation at 710 nm and emission at 760 nm) injected systemically at a dose of 10 million cells per mouse. Fluorescence image acquisition was performed at 24, 48, and 72 h after BMC2-CBG2 macrophage administration. The respective radiant efficiencies were quantified and plotted. Bioluminescence photon fluxes represent the light emission for B16-CBR2 melanoma before BMC2-CBG2 macrophage administrations. BL images were acquired 15 min after NH_2_-NpLH2 substrate injection. The bar graph shows the mean fluorescence intensity (*n* = 3 mice). Significance was registered for mean values of 24- and 48-h groups versus mean values of the 72-h group. (*) *p* < 0.05. Error bars represent ±SD.

To investigate the potential of BMC2 macrophage sensors as a tool for melanoma detection, C57BL/6 mice were subcutaneously engrafted with B16F10 melanoma cells expressing CBR2 luciferase (B16-CBR2). Tumor growth was spectrally imaged using the NH_2_-NpLH2 luciferin substrate and monitored over time by BL imaging. To set up the optimal cell concentration and optimal time-point for the detection of DiR-labeled BMC2-CBG2 macrophages, we first injected DiR-labeled BMC2-CBG2 macrophages systemically at three cell amounts: 1 × 10^6^, 5 × 10^6^, and 10 × 10^6^ and 2 weeks after subcutaneous melanoma implantation. Fluorescence values for DiR-labeled BMC2-CBG2 macrophages were collected after BLI detection of B16-CBR2 melanoma tumors, and fluorescence values are plotted in Figure 2A. The proportional correlation between the number of DiR-labeled BMC2-CBG2 cells injected and the radiant efficiency registered show that both five and 10 million DiR-labeled BMC2-CBG2 cells can be efficiently measured in subcutaneous tumors (Figure 2A). Interestingly, the accumulation of DiR-labeled BMC2-CBG2 macrophages adjacent to the non-tumor areas is probably due to the on-target recruitment of macrophages with poor tumor-infiltration capability (Figure 2A, right panel). To validate the feasibility to visualize the DiR-labeled BMC2-CBG2 macrophages in subcutaneous tumor models, we first measured the BL photon yields in mice bearing wildtype B16-CBR2 melanoma tumors, and later, we injected the DiR-labeled BMC2-CBG2 cells with three different cell numbers: 1 × 10^6^, 5 × 10^6^, and 10 × 10^6^. BL outputs from the DiR-labeled BMC2-CBG2 macrophages were registered after NH_2_-NpLH2 substrate administration and selecting appropriate bandpass filters ranging from 580 to 800 nm. Dim photon fluxes (∼4 × 10^4^ photon/s) were registered for the wildtype B16-CBR2 melanoma-bearing mice after receiving 10 million BMC2-CBG2 cells (Supplementary Figure S1A). Additionally, BL outputs for BMC2-CBG2 cells resulted insufficient to register an accurate spectral library and to perform a spectral unmixing algorithm. On the contrary, near-infrared FL outputs of BMC2-CBG2 cells were accurately measured at the subcutaneous tumor area allowing more sensitive detection of the DiR-labeled BMC2-CBG2 macrophages compared to BL imaging. However, FL imaging of the DiR-labeled BMC2-CBG2 macrophages confirmed colocalization with the dim BL outputs found in the tumor region (Supplementary Figure S1A). Interestingly *ex vivo* data demonstrated that the highest value for DiR fluorescence from labeled BMC2-CBG2 macrophages was substantially detected in the lungs (1,6E+10 [ph/s/cm^2^/sr]/[μW/cm^2^]). The DiR fluorescence of subcutaneous melanoma registered instead radiant efficiency value that was ∼7,3 fold lower compared to the DiR values registered in the lungs (Supplementary Figure S1B right panel). Once we assumed that 10 million BMC2-CBG2 cells gave the most accurate FL and BL signals *in vivo*, we further investigated the optimal time-point for imaging the DiR-labeled BMC2 cells in subcutaneous melanoma models. B16-CBR2 tumor-bearing mice were routinely imaged to monitor melanoma cancer growth by BLI (Figure 2B). Two weeks after tumor implantation, 10 million DiR-labeled BMC2-CBG2 cells were injected systemically, and FL measurements were performed at 24, 48, and 72 h post BMC2-CBG2 administration on the IVIS spectrum imager (Figure 2B). Control mice received B16-CBR2 melanoma cells only (data not shown). Radiance values at 72 h after DiR-labeled BMC-CBG2 injection were ∼3 fold higher than the radiance values registered at 24 and 48 h at the melanoma tumor site. (Figure 2B). *Ex vivo* near-infrared FL imaging confirmed the localization of DiR-BMC2 macrophages at the subcutaneous tumor area at 72 h after DiR-labeled BMC2-CBG2 injection (Supplementary Figure S2A, right panel). Control mice were inoculated with B16-CBR2 melanoma tumors only. Together, the data suggest that the optimal time-point to localize 10 million DiR-labeled BMC2-CBG2 macrophages at the subcutaneous melanoma tumor site is 72 h after DiR-labeled BMC2-CBG2 macrophage administration.

### Engineered BMC2 Macrophages Enable *In Vivo* Visualization of Melanoma Lung Metastasis by Bioluminescence and Fluorescence Imaging

**FIGURE 3:**
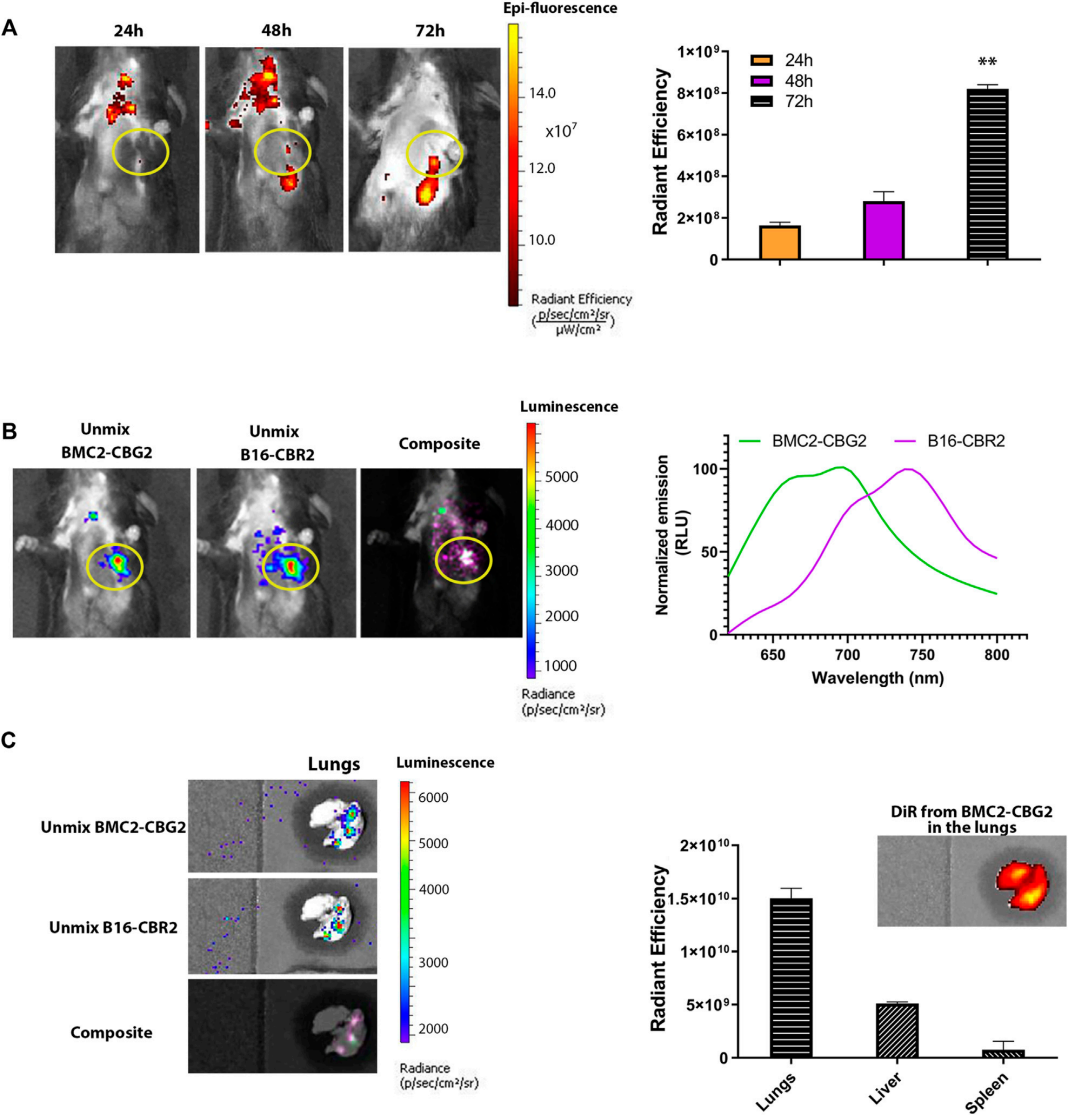
Engineered macrophages enable *in vivo* visualization of the metastatic tumor model by BLI. **(A)** Fluorescence imaging of mice bearing B16-CBR2 metastatic cancer cells (*n* = 3 mice). After metastatic tumor establishment, C57BL/6 mice were injected with 10 million BMC2-CBG2 macrophages pre-labeled with the DiR near-infrared dye. Fluorescence imaging was performed at 24, 48, and 72 h post BMC2-CBG2 administration (left panel). Fluorescent outputs registered from the lungs were recorded and plotted in the right panel, demonstrating that BMC2-CBG2 macrophages accumulate at the tumor site mainly at 72 h after BMC2-CBG2 macrophage administration. The ROI region for the tumor site is marked in the yellow circle. The bar graph shows mean fluorescence intensity (*n* = 3 mice). Significance was registered for mean values of 24- and 48-h groups versus mean values of the 72-h group. (*) *p* < 0.05 was calculated by one-way ANOVA followed by Tukey’s test. Error bars represent ±SD. **(B)** Representative BL spectral unmixing of a mouse engrafted with metastatic B16-CBR2 melanoma (*n* = 3) (left panel). Mice (*n* = 3) were imaged 48 h after the injection of 10 million BMC2-CBG2 macrophages pre-labeled with the DiR near-infrared dye. Images were acquired 15 min after NH_2_-NpLH2 substrate injection. The filter selected for the green spectral unmixing (BMC2-CBG2) was set at 700 nm, and for the magenta spectral unmixing (B16-CBR2), the filter was set at 740 nm. Spectral properties of BMC2-CBG2 macrophages and B16-CBR2 melanoma with the NH_2_-NpLH2 substrate are depicted in the right panel. The ROI region for the tumor site is marked in the yellow circle. **(C)** Representative images of *ex vivo* spectral unmixing of the lungs. The green color was used for unmixed BMC2-CBG2 macrophages and magenta color for unmixed B16-CBR2 melanoma tumors. Colocalized bioluminescent signals for BMC2-CBG2 and B16-CBR2 cells in the lungs resulted in overlapped colors (light pink), as shown in the composite image (left panel). Radiant efficiency values for DiR-labeled BMC2-CBG2 macrophages were measured in the lungs, livers, and spleens and are depicted in the right panel. FL emissions for the DiR fluorescent dye were recorded from the lungs, liver, and spleen, selecting the DiR filters for excitation at 710 nm and emission at 760 nm. The bar graph shows the mean fluorescence intensity. Error bars represent ±SD.

We further explored whether BMC2-CBG2 macrophages may be used as a diagnostic cell sensor in metastatic cancer models. Thus, orthotopic metastatic C57BL/6 mouse models were established with systemic injections of B16-CBR2 cells. Tumor growth was monitored over time by BL imaging of CBR2 luciferase after administration of the NH_2_-NpLH2 luciferin substrate. Once the tumor growth could be visualized by BLI and showed spreading in the chest (usually on day 10), DiR-labeled BMC2-CBG2 macrophages were injected systemically (1 × 10^7^ cells per mouse). Fluorescence outputs for DiR-labeled BMC2-CBG2 macrophages were calculated by drawing the region of interest (ROI) at the metastatic tumor region (yellow circle). DiR radiance values were collected at 24, 48, and 72 h after BMC2-CBG2 macrophage injection (Figure 3A), considering that BMC2-CBG2 macrophages could retain the DiR *in vitro* at least 72 h (Supplementary Figure S3A). A substantial localization of DiR-labeled BMC2-CBG2 macrophages was registered in the metastatic lungs at 72 h post macrophage injection (Figure 3A). Interestingly, FL imaging measured a strong localization of DiR-labeled BMC2-CBG2 cells also in the cervical lymph node at 24 and 48 h post injection of macrophages. Radiant efficiencies of the DiR dye measured in the metastases at the three different time points are depicted in Figure 3A (right panel). In addition, we successfully applied the spectral unmixing algorithm to separate the colocalized bioluminescent signals of BMC2-CBG2 and B16-CBR2 in the lungs (Figure 3B). To perform that, specific bioluminescent spectral libraries for pure BMC2-CBG2 and B16-CBR2 outputs were built. Of note, BL outputs emitted by either BMC2-CBG2 (green color) or B16-CBR2 (magenta color) were accurately extracted from the melanoma area 48 h post BMC2-CBG2 administration (Figure 3B). The spectral properties of BMC2-CBG2 and B16-CBR2 were measured and depicted in Figure 3B (right panel). The spectral unmixing algorithm was also conducted for *ex vivo* harvested lungs 72 h post BMC2-CBG2 administration. The representative images of BL spectral unmixing are shown in Figure 3C. Radiant efficiency values for DiR pre-labeled BMC2-CBG2 macrophages were measured in the lungs, livers, and spleens. FL outputs were plotted highlighting that the values for the DiR registered in the lungs were 2-3 fold greater than the values in the liver.

To confirm the localization of the activated BMC2-CBG2 macrophages to the tumor site, 15-micron thick tumor sections from mice inoculated with DiR-labeled BMC2-CBG2 cells were stained for CD68 to detect macrophages, as shown in red color in Supplementary Figure S3B.

Furthermore, confirmation of injected macrophages labeled with the DiR dye was conducted by imaging scan of cryosections of the lungs infused with DiR-labeled BMC2-CBG2 macrophages using the Odyssey CLx device. Filters at 700 and 800 nm were selected for the imaging. A channel at 700 nm was used to distinguish CD68+ macrophages, and a channel at 800 nm was used to distinguish macrophages labeled with the DiR dye. A substantial difference was detected between B16 tumors treated with DiR-labeled BMC2-CBG2 macrophages localized at the tumor site (green color, top panels) compared to the B16 tumor controls not treated with DiR-labeled BMC2-CBG2 macrophages (bottom panels) (Supplementary Figure S3C).

Together, these data suggest the feasibility to visualize and localize DiR-labeled BMC2-CBG2 macrophages in the lungs affected by metastatic melanoma tumors.

## Discussion

The timely detection of cancer growth, cancer recurrence, and monitoring therapies will increase the chances of prompt response to medical intervention. The success of blood-based biomarkers for early tumor lesions is often limited by short circulation times, blood dilutions, and demonstrating suboptimal sensitivity for cancer diagnostics (Mamdani et al., 2017). Encouraging the use of engineered immune cells as an emerging class of cellular sensors for inflammation and disease will foster new technologies for cellular immunotherapies. A clear advantage of using immune cells such as macrophages as diagnostic vehicles is based on their homing and infiltrating capabilities at the cancer sites (Muthana et al., 2013). Recently, Aalipour A. and coworkers in a seminal work, exploited macrophages engineered to express the Gluc luciferase gene activated when switching their phenotype in tumor-associated macrophages (TAMs) in the tumor area. The BL measurement of the Gluc activity was conducted by a simple blood test for sensitive detection of modest breast cancer tumor volume (25–30 mm^3^) (Aalipour et al., 2019).

Melanoma tumor is intrinsically linked to an inflammation reaction and therefore stimulates the recruitment of “tumor-homing” cells such as macrophages. Thus, we leveraged the natural property of macrophages to infiltrate subcutaneous and metastatic melanoma tumors and image them by dual-color BLI and by near-infrared FL *in vivo*. In our case, *in vivo* and *ex vivo* data showed that we could visualize macrophages homing small metastatic melanoma tumors in the lungs (size ranging between 30 and 100 mm^3^) 15 days after tumor inoculation. To perform sensitive real-time dual-color bioluminescence imaging in deep tissues, we engineered BMC2 macrophages to express the CBG2 luciferase mutant (*λ* = 680 nm) and B16F10 melanoma cells to express the CBR2 click beetle luciferase mutant (*λ* = 740 nm) (Hall et al., 2018; Zambito et al., 2020). We applied this technology using NH_2_-NpLH2 luciferin as a single substrate to monitor immune cell dynamics and tumor growth qualitatively. The administration of a unique substrate makes the BLI sessions highly specific compared to other systems where multiple injections of substrates are required (Hall et al., 2018; Ji et al., 2020; Zambito et al., 2020). However, we expect the performance of dual-color BLI using orthogonal luciferase/substrate couples such as AkaBLI (Iwano et al., 2018) and NanoLuc/hydrofurimazine systems (Su et al., 2020) would be similar. Moreover, our approach can also be exploited to genetically engineer various tumors and immune cells such as T cells, natural killer cells, and dendritic cells.

In the murine orthotopic melanoma model presented here, BMC2-CBG2 macrophages exhibited a visible accumulation in the primary tumors at 72 h after systemic administration. However, substantial off-target cell sequestration was also observed in the liver and the spleen. These findings were supported by the work of Combes F. and others, where viable DiR-labeled macrophages were first partly sequestered in the lungs and then redistributed to other off-target sites such as the liver and the spleen and also to on-target tumor sites up to 96 h after systemic administration (Combes et al., 2018).

The cell kinetics observed in this study are also supported by seminal imaging studies on the migratory properties of radio-labeled macrophages homing tumors by PET imaging modality. The biodistribution of engineering macrophages pre-labeled with an ^18^F-FDG probe (half-life = 109.7 min) proved that macrophage-activated killer (MAK) cells are first sequestered in the liver and lungs and to a minor degree in the spleen after i.v. injection of patients. Then, cell redistribution occurs from the pulmonary vasculature to other tissues including peritoneal metastases in human patients at 4 h after ^18^F-FDG-labeled macrophage-activated killer (MAK) cell administration. However, the short-life of ^18^F-FDG and “leakage” of the probe out of the cells should be considered when performing cell biodistribution studies (Ritchie et al., 2007). In another study, also SPECT tracking of i.v. injected indium-oxine (half-life = 2.8 days)–labeled murine macrophages reveals the initial margination of macrophages in the lungs and then eventual migration to the liver, spleen, and kidneys before accumulating within sites of the tumor (Chokri et al., 1992). An improved alternative to future clinical application would be employing the ^89^Zr-oxine probe that has a longer half-life (3.3 days), and it has been largely used to track infused T cells homing breast cancer and imaged for several days by PET imaging modalities in small animals (Man et al., 2019; Kurebayashi et al., 2021). However, to the best of our knowledge, ^89^Zr-oxine has not been used to track macrophages *in vivo* yet. Additionally, the accumulation of macrophages in off-target organs should not be discounted when considering the use of macrophages as a cell-based platform for tumor diagnostic or the delivery of drugs into tumors and will be part of a follow-up study.

In our study, an adequate accumulation of macrophages was measured and visualized in subcutaneous and metastatic tumor models together with the longitudinal tracking of immune cells by bioluminescence. The fact that we could image macrophages by *in vivo* bioluminescence also suggests the accumulation of living cells at the tumor site and, therefore, excludes labeling artifacts attributed to the fluorescence observed.

Overall, we envisioned that engineered macrophages should be considered for their innate ability to sense and reach small tumors and metastasis in the body. Moreover, the advancement of optical imaging technologies and the design of novel near-infrared I and II probes will encourage to shed light on the mechanisms underlying macrophage recruitment and behavior in animal models, especially in genetically-engineered mouse models with spontaneous tumor formation.

## Data Availability

The raw data supporting the conclusions of this article will be made available by the authors, without undue reservation.
