# Supplementary figures and images for: Near-Infrared Bioluminescence Imaging of Macrophage Sensors for Cancer Detection In Vivo
Source: Front Bioeng Biotechnol. 2022 May 9;10:867164. doi: 10.3389/fbioe.2022.867164 (PMC9124759; doi:10.3389/fbioe.2022.867164)

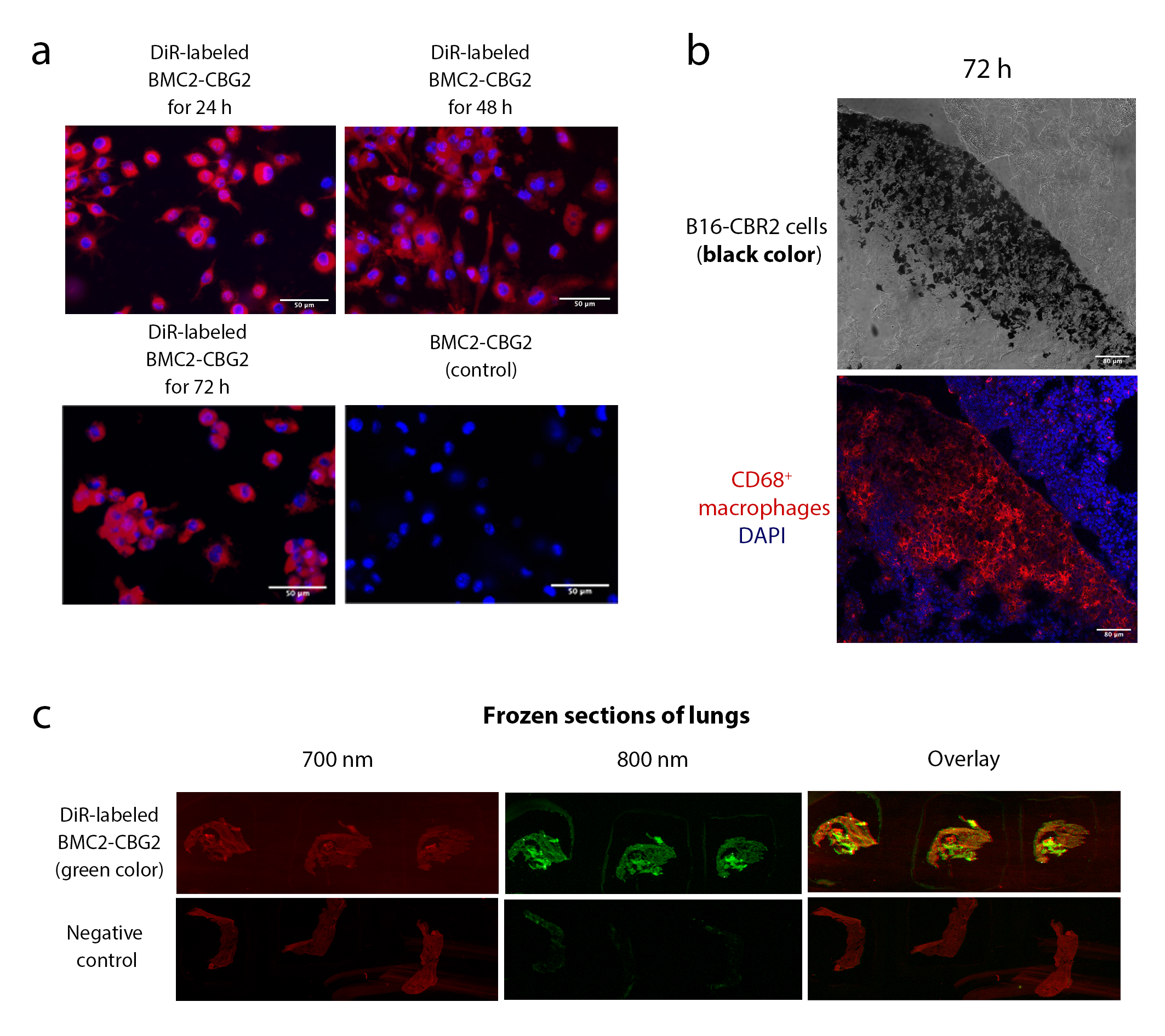

Supplement: Supplementary file 1 [file Image3.TIF]

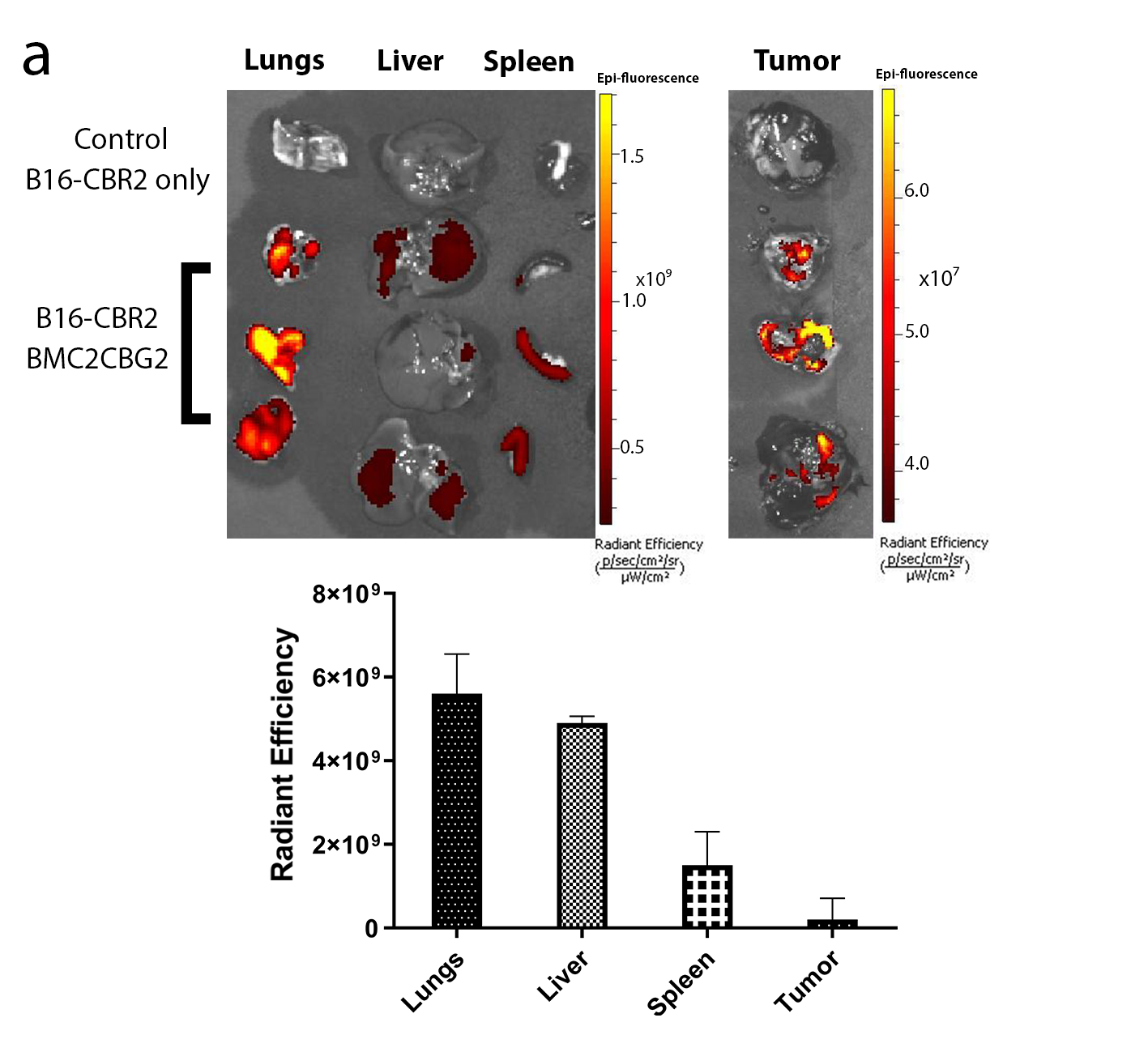

Supplement: Supplementary file 2 [file Image2.TIF]

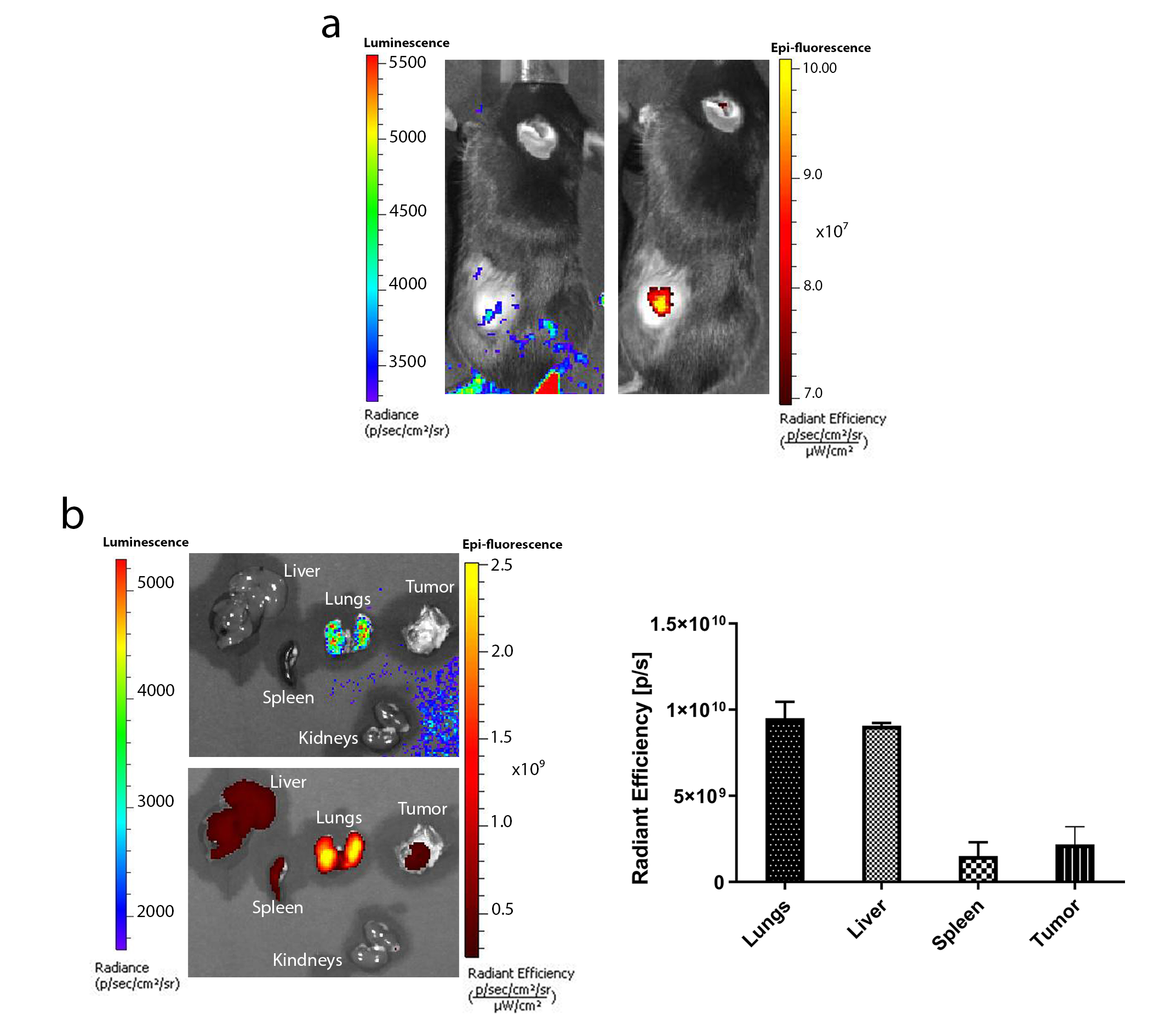

Supplement: Supplementary file 3 [file Image1.TIF]
